# *Corky*, a *gypsy*-like retrotransposon is differentially transcribed in *Quercus suber* tissues

**DOI:** 10.1186/1756-0500-5-432

**Published:** 2012-08-13

**Authors:** Margarida Rocheta, Luísa Carvalho, Wanda Viegas, Leonor Morais-Cecílio

**Affiliations:** 1 Centro de Botânica Aplicada à Agricultura (CBAA), Departamento de Recursos Naturais, Ambiente e Território, Instituto Superior de Agronomia, Universidade Técnica, de Lisboa, PORTUGAL

**Keywords:** *Quercus suber*, *Corky*, LTR retroelement, RT-qPCR, Expression

## Abstract

**Background:**

Transposable elements (TEs) make up a large part of eukaryotic genomes. Due to their repetitive nature and to the fact that they harbour regulatory signals, TEs can be responsible for chromosomal rearrangements, movement of gene sequences and evolution of gene regulation and function. Retrotransposon ubiquity raises the question about their function in genomes and most are transcriptionally inactive due to rearrangements that compromise their activity. However, the activity of TEs is currently considered to have been one of the major processes in genome evolution.

**Findings:**

We report on the characterization of a transcriptionally active *gypsy*-like retrotransposon (named *Corky*) from *Quercus suber*, in a comparative and quantitative study of expression levels in different tissues and distinct developmental stages through RT-qPCR. We observed *Corky*’s differential transcription levels in all the tissues analysed.

**Conclusions:**

These results document that *Corky*’s transcription levels are not constant. Nevertheless, they depend upon the developmental stage, the tissue analysed and the potential occurring events during an individuals’ life span. This modulation brought upon by different developmental and environmental influences suggests an involvement of *Corky* in stress response and during development.

## Background

Retrotransposons are generally the most abundant class of Transposable Elements (TEs), concerning their proportion in the genomes and, are widely distributed among eukaryotic genomes, especially in plants [1]. Due to their wide distribution and the diverse types of induced mutations, TEs are thought to have contributed significantly to eukaryotic genes and genomes evolution [2]. The increasing number of data obtained from genome-wide sequencing projects indicate that TEs take part in major events and are a potential pool of promoter regions for host regulatory sequences [3]. TE regulatory regions are known to be sequences of extremely rapid evolution, a characteristic of eukaryotic regulatory regions attributed to having to cope with changing genomic environments [4]. LTR-retrotransposons are 'copy-and-paste' (class I) TEs that replicate via an RNA intermediate. Like animal retroviruses, these retrotransposons have two LTRs, with signals for transcription initiation and termination, flanking an internal region (gag-pol) that typically contains genes and other features necessary for autonomous retrotransposition. Retrotransposon ubiquity raises the question about their function in genomes. Retrotransposon insertions in, or next to coding regions, generate mutations that can lead to changes in gene expression. For instance, *Tnt*1A transposition preferentially targets genic regions, suggesting that the activity of transposable elements can modulate genic functions and represent a natural source of phenotypic diversity [5]. Furthermore, run-off transcription from retrotransposons can lead to overexpression or suppression of nearby genes [6]. Transcription activity detected in several retrotransposons during certain stages of development seems to point to a potential role of these elements during plant growth [7,8]. Additionally, some biotic and abiotic stresses can increase transcript levels of retroelements, such as tobacco *Tnt1*[9], *Tto1*[10], *Tto2*[11], rice´s *Tos17*[12] and *Rtsp-1* from sweet potato [7]. An overall picture of retrotransposon expression is however difficult to establish due to the absence of exhaustive comparative studies in different tissues. Several *Gypsy* and *Copia-*like retroelements are known to be well represented in the Mediterranean *Quercus suber*[13,14].

*IFG*7 [15] is one of the most representative *Gypsy*-like elements in coniferous genomes such as in several Pines [15-18]and *Taxodium distichum*[19], and sometimes is considered as a conifer-specific LTR retroelement [20]. However, elements like *IFG*7 were not yet identified in Angiosperms. In order to study the possible occurrence of a conifer derived LTR retroelement in a distant related Angiosperm tree species, as well as its potential active transcriptional activity in this species, we used *IFG*7 as a *Gypsy* representative element.

The key aims of this work were the molecular characterization of a new retrotransposon in the *Quercus suber* genome which is homologue to the previously identified *IFG*7 from *Pinus radiata*[15] and *PpRT*1 from *Pinus pinaster*[18] and, the quantification of its transcriptional activity in different tissues and distinct developmental stages and conditions. Together, the data presented here clearly show that this retrotransposon, named *Corky*, makes up a dynamic component of the cork oak genome.

## Findings

### Organization and structure of *Corky*

*Corky* is a *gypsy* retroelement that was isolated throughout genome walking in *Q*. *suber* genome. All generated DNA fragments were sequenced and further analysed.

**Figure 1:**
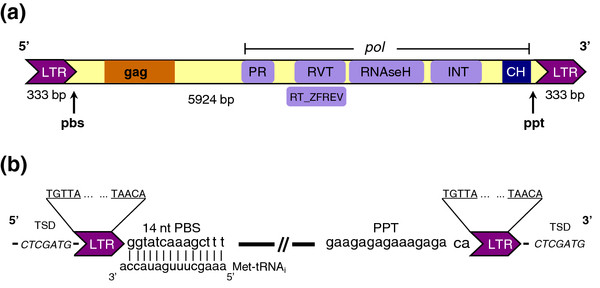
**Organization and structure of *Corky* retrotransposon.** (**a**) LTR, long terminal repeat; PR, protease; RVT, reverse transcriptase; R, RNAse H; INT, integrase; CH, chromatin organization modifier. PBS, primer binding site. PPT, polypurine tract. The entire element has a length of 5924 bp with 333 bp LTRs. (**b)** The terminal boxes at each end of *Corky* represent LTRs. The Target Site Duplications (TSD) of 7 bp direct repeat flanking *Corky* is shown. Above the element, the 5 bp inverted repeats (5’­TGTTA…TAACA-3’) within each LTR are shown in the expansions. The primer binding site (PBS) and polypurine tract (PPT) are boxed. 14 nucleotides of the 3’ end initiator methionine tRNA complementary to the PBS region of *Corky* is shown.

The assemblage of all the sequences revealed that this retrotransposon is 5924 bp long (GeneBank: EU862277) (Figure 1a) and harbours internal regions with homology to retroviral genes *gag* and *pol*. The *pol* region contains sequence motifs related to the enzymes protease, reverse transcriptase, RNAseH and integrase in the same typical order known for *gypsy*-like retrotransposons. The complete sequence analysis reveals that the reverse transcriptase (RVT), RNaseH and integrase (INT) have the same nucleotide number as *PpRT*1 [18] with nucleotide identity percentage of 92%, 96% and 95%, respectively. Additionally, the HPVFH(V)S integrase motif in *Corky* is distinct from HLVFH(D)S found in *PpRT*1 and *IFG*7 retrotransposons. Two substitutions occurred in *Corky*: a leucine to a proline and an aspartic acid to a valine (Additional file 1). Changes in these motifs might be responsible for the specific targeting and insertion [21]. Flanking the 3’LTR, another region was identified as a chromatin organization modifier (CH) [22], with 50 amino acids, which appear to play a role in the functional organization of the eukaryotic nucleus and probably targets the element to regions of high gene expression [23].

Each LTR is 333 bp long and is flanked by a short 7 bp direct repeat 5’- CTCGATG-3’ (Figure 1b), probably representing a duplication of the genomic target site produced by the insertion of a *Corky* copy, such as it has been reported for other retroelements [24]. Both LTRs begin and end with a 5 bp inverted repeat 5’­TGTTA…TAACA-3’ including the retroviral consensus 5’-TG…CA-3’. LTRs inverted repeats are present in all retroviruses and are thought to be important for their integration [25] (Figure 1b). 5’LTR*´s Corky* sequence analysis (Additional file 2) revealed two characteristic patterns of repeating motifs: one is a simple pattern of short tandem sequence motifs (a..a..) TA(G)TGATTACCCC(A)T(T)(A) and TA(T)TG(T)ATTA(TA)CCCC(T)T(A)(T), while the other one, more complex, has two adjacent heterologous motifs (TATTGTTA, TTATATT), repeated twice as a group (ab..ab), as present within the *HIV*-1 and *gypsy* enhancers [26]. Both patterns are dispersed between the two TATA sequences (TATATATA) (Additional file 2). Enhancers typically consist of a series short repeated sequence motifs that are often associated with regulatory protein binding domains [27].

### Quantification of *Corky* expression

**Figure 2:**
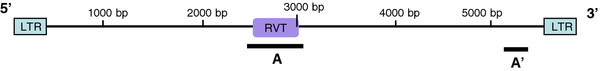
**Fragments position in *Corky* element used in RT- qPCR.** Real-time PCR was performed in two regions of *Corky* element: RVT (**A**) and the region between the integrase and the chromodomain **(A’).**

**Figure 3:**
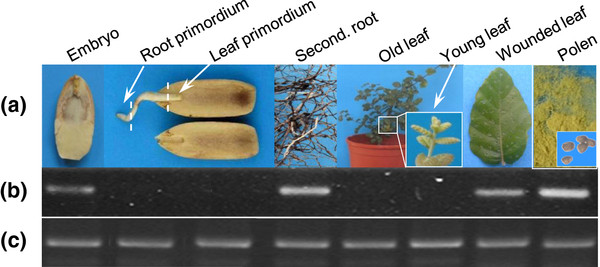
**RT-qPCR analysis of *Corky* transcriptional activity.** (**a**) Several tissues and developmental stages *from Quercus suber*. (**b**) RT-qPCR amplification of *Corky* RVT produced a single band of 636 bp with different quantity levels. (**c**) RT-qPCR amplification of *actin* produced a unique band with 350 bp and with the same intensity in all tissues.

*Corky’s* transcription levels were monitored using the RVT and a region between the integrase and the chromodomain (Figure 2) in ten replicates of several tissues and developmental stages: embryos, root and leaf primordia (15 days after seed germination), secondary roots, old and young leaves (intact and wounded) from 2.5 year old trees and pollen grains using RT-qPCR (Figure 3).

**Figure 4:**
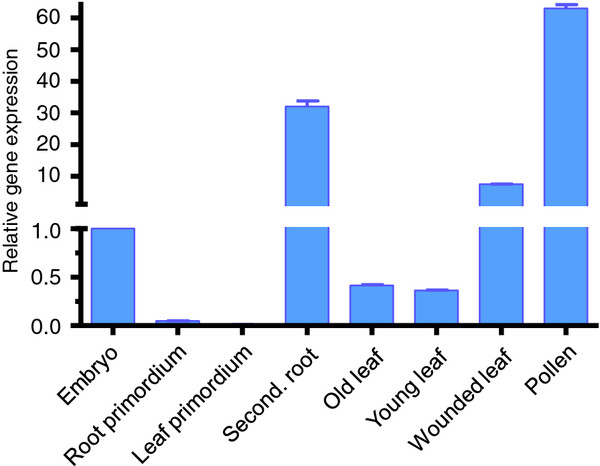
**Relative *Corky* expression quantified through RT-qPCR in different tissues.** mRNA was isolated from each tissue, converted to cDNA, and subjected to RT-qPCR. Relative amounts were calculated and normalized with respect to *actin* mRNA. Values are expressed as fold variation of each tissue type relative to the embryo (control, expression = 1).

The results obtained for both *Corky* regions revealed to be similar. Transcripts quantification throughout plant development, clearly demonstrated that this retroelement is always active although with significant difference between organs/cells (Figure 3 and 4). The highest *Corky* expression was detected in pollen, usually exposed to high levels of stress represented by an extremely low cell hydration state. High levels of expression were also detected in secondary roots (Figure 4). This situation can be interpreted in a developmental point of view, considering that the meristematic activity leading to root expansion increases the levels of *Corky* transcription, as it was already detected [28]. Furthermore, *Corky*’s high levels of expression could also be due to potential wounding caused by roots growing through soil, as has also been reported for *TLC1* in tomato [29] and *Cire*1 in sweet orange [8]. The association of *Corky* activity with stress is even stronger when healthy leaves are compared with those subjected to a mechanical stress similar to herbivory, increasing the number of transcripts (Figure 4). When we compared embryos, in a dormancy state, with two regions (root and leaf primordia) of the same embryo in the initial steps of germination we found high levels of transcript in the first condition, probably because in regions with high levels of cell division retrotransposon expression is not required. These results revealed that *Corky* expression is not only associated to stress conditions but also to different developmental stages. Taken together, these findings suggest that *Corky* has escaped from host silencing mechanisms and might have been preserved to a potential selective advantage.

## Conclusion

Our data show good evidence that a retrotransposon (*Corky*) has escaped from host silencing mechanisms. The differential expression in several plant tissues in different developmental stages suggests, at least, an involvement of this retrotransposon in stress response and in developmental processes. It is likely that retroelements do not increase plasticity in an evolutionarily active way but they might play a crucial role in response to developmental/environmental challenges. Together, these results set the need to further investigate both regulation and control mechanisms that implicate retrotransposons and development.

## Materials and methods

### Plant material

Acorns of *Quercus suber* L. produced by open pollination and pollen used in this study were collected in a natural population at Alcácer do Sal (Portugal). The plants used in this study were obtained from those acorns and grown in the greenhouse until they were used (at 2.5 years old). Plant tissues were frozen in liquid nitrogen. Genomic DNA was extracted from samples using DNeasy® Plant Mini Kit (Qiagen®), according to the manufacturer’s instructions.

### Initial DNA amplification strategy

The first set of primers [Forward- 5’ttcaactgagtcaaatttc3’ and Reverse- 5’ctgtcaacccaagaaatcctcgcag 3’] (Additional file 3) used, were constructed by the assumption that the RVT sequence in *Q. suber* has sufficient similarities with the previous retrotransposon amplified in *P. pinaster* (named *PpRT*1) [18]. For this part of the work only DNA from young leaves was handled. A set of primers was designed to guarantee that we are in the presence of the same copy of *Corky* (Additional file 3). The PCR protocol consisted of the subsequent steps: an initial denaturation period at 94°C for 4 min., 30 cycles of amplification, each of which consisted of 45 s of denaturation at 94°C, 45 s of annealing at 57°C, and 90 s of elongation at 72°C with a final elongation step of 4 min at 72°C. After purification with the QIAquick® PCR purification kit, the amplified fragment was cloned using pCR 2.1-TOPO vector (Invitrogen®) and sequenced.

### Genome walking

Genome walking was performed using the Genome Walker® kit (Clontech®) components according to the manufacturer’s instructions. The amplification of upstream and downstream regions of RVT sequences from the libraries was performed also according to the Genome Walker® Kit protocol and the primers melting temperature (Additional file 3). All the PCR amplifications were performed with the proofreading enzyme Phusion (New England Biolabs®). The major PCR products obtained were gel extracted by the Gel Extraction® Kit (Qiagen®) additionally inserted in pCR 2.1-TOPO vector® (Invitrogen®), sequenced and aligned using the online service of National Center for Biotechnology Information (NCBI) [30]. To guarantee that all sequences belong to the same retroelement we performed numerous amplifications for the same region with different sets of primers. Additionally, primers were designed assuring that all fragments amplified overlap. Thus, all the fragments obtained were used to assemble the entire retroelement. Conversely, without other resources such as Bacterial Artificial Chromosomes (BACs), we cannot say that we have isolated the same genomic element. Although, the high overlap of the individual sequences ensures that we have got the same element, we cannot discard the hypothesis that we have reconstructed a chimeric sequence. The assembled sequence was used to search all the retrotransposon regions between both LTRs, according to the conserved motives.

### RNA isolation and cDNA preparation for RT-qPCR

Total RNA was extracted from secondary roots, old leaves (one year old) and young leaves (from the year) from ten 2,5 year old plants, from ten dormant embryos, from the primordia of leaves and roots of ten germinated embryos and from ten different pollen samples, each replicate corresponding to tissue originating from one single plant and also from ten wounded leaves (leaves were pierced with a needle 240 min prior to freezing), using the RNAqueous® kit (Ambion®), according to the manufacturer’s instructions. Nucleic acid concentration of each sample was quantified by spectrophotometry using the software Gen5 1.09 (Synergy HT, Bio-Tek Instruments, Winooski, USA). Total RNA quality was assessed by the A_260_/A_280_ and A_260_/A_230_. Only RNA samples with A_260_/A_280_ between 1.8 and 2.1 and A_260_/A_230_ between 2.0 and 2.2 were accepted for the experience. Total RNA integrity was tested through 1% agarose gel electrophoresis under denaturing conditions.

RNA samples were treated with RQ1 RNase-Free DNase (Promega, Madison, WI). cDNA was synthesized from 2 μg of total RNA using random hexamers and Superscript II RNase H- reverse transcriptase (Invitrogen®, Carlsbad, CA), according to the manufacturer’s recommendations followed by PCR amplification using specific primers for the RVT and a region between Integrase and the chromodomain of *Corky* (Figure 2). As expected, amplification products were not obtained in RNA samples not yielded to reverse transcription prior to PCR. cDNA was stored at −20°C.

### Transcriptional activity of *Corky*

RT-qPCR was performed in a 96 well white reaction plates (Bio-Rad®, Hercules, CA), using an IQ5 Real Time PCR (Bio-Rad®, Hercules, CA) with ten biological replicates and two technical replicates. For amplification specific primers corresponding to the RVT domain of *Corky* and a region between the Integrase and the chromodomain were used (Figure 2). Each 20 μL reaction mixture well contained 10.0 μL of 2x master mix iQ SYBR Green Supermix®, 2.0 μL of HPLC-purified primers (10 μM), 7.0 μL of PCR-grade H_2_O and 1.0 μL target DNA solution. PCR amplification products were monitored via intercalation of SYBR-Green (included in the master mix). The PCR protocol consisted of an initial denaturation step at 95°C for 3 min, 40 cycles of amplification, each of which consisted of 15 s of denaturation at 95°C, 20 s of annealing at 57°C and 50 s of elongation at 72°C. As expected, amplification products were not obtained in RNA samples not subjected to the reverse transcription step prior to PCR.

To assess the primers amplification efficiency, identical volumes of cDNA samples were diluted and used to generate five-point standard curves based on a five-fold dilution series (1; 1:5; 1:25; 1:125; 1:625), in triplicate. Amplification efficiency (E) is calculated as E = 10^(−1/a)^-1, “a” being the slope of the linear regression curve (y = a log (x) + b) fitted over the log-transformed data of the input cDNA dilution (y) plotted against the respective quantification cycle (Cq) values (x). E-values of the target genes were considered comparable when they did not exceed 100 ± 10%, corresponding to a standard curve slope of 3.3 ± 0.33. All cDNA samples were diluted 50 fold and were amplified in duplicate in two independent PCR runs.

To generate a baseline-subtracted plot of the logarithmic increase in fluorescence signal (ΔRn) versus cycle number, baseline data were collected between the cycles 5 and 17. All amplification plots were analysed with an *R*_*n*_ threshold of 0.2, at the beginning of the region of exponential amplification, to obtain Cq and the data obtained were exported into a MS Excel workbook (Microsoft® Inc.) for further analysis. In order to compare data from different PCR runs or cDNA samples, C_q_ values were normalized to the C_q_ value of *actin*, a housekeeping gene expressed at a relatively high and constant level [31]. Gene expression was calculated using the ΔΔC_q_ method [32]. Results are expressed as fold variation of each tissue relative to each of the other.

## Abbreviations

C_q_: threshold cycle; bp: base pairs; nt: nucleotides; RT-qPCR: reverse transcription real time PCR; TE: transposable element; RVT: reverse transcriptase.

## Competing interests

The authors declare that they have no conflict of interest.

## Authors’ contributions

MR and LM conceived the experiments; MR and LC performed the experiments; MR, LM and LC analysed the data and wrote the manuscript; WV commented the manuscript. All authors read and approved the manuscript.

## Supplementary Material

Additional file 1 **Structural features of  *Corky* retrotransposon.** Conserved amino acid (single letter code) domains of Reverse Transcriptase (underline), RNaseH, Integrase (underline) and Chromo (underline). In the Reverse Transcriptase two important motives PFGL and DDILIYS are in red. In RNaseH the CDAS motif is pointed in bold. In the integrase the three subdomains are in red: HH-CC; D,DE and G-(D/E)-X_10-20_-KL-*X*_2_/R/K)-F/Y/W)-X-G-P-(F/Y)-X-(I/V). The HPVFH(V)S motif is showed in bold. Click here for file

Additional file 2 **Nucleotide sequence of the 5’ LTR from *Corky* **. The repeating sequences motifs are underlined and the two TATA box sequences are boxed. Click here for file

Additional file 3 **Primers used to amplify  *Corky* **. Click here for file
