# Supplementary material for: Corky, a gypsy-like retrotransposon is differentially transcribed in Quercus suber tissues
Source: BMC Res Notes. 2012 Aug 13;5:432. doi: 10.1186/1756-0500-5-432 (PMC3465219; doi:10.1186/1756-0500-5-432)
Supplement: Additional file 1 — Structural features of Corky retrotransposon. Conserved amino acid (single letter code) domains of Reverse Transcriptase (underline), RNaseH, Integrase (underline) and Chromo (underline). In the Reverse Transcriptase two important motives PFGL and DDILIYS are in red. In RNaseH the CDAS motif is pointed in bold. In the integrase the three subdomains are in red: HH-CC; D,DE and G-(D/E)-X10-20-KL-X2/R/K)-F/Y/W)-X-G-P-(F/Y)-X-(I/V). The HPVFH(V)S motif is showed in bold. [file 1756-0500-5-432-S1.pdf]

5’…LVHEKDGSWCMCLDYRELNKLTIKDKFPIPVIDELLDELHGSIYFTKLDLR **RVT**
SGYHQIRMKMKDILKTTFRTHEGHYEFLVM**PFGL**TNAPSTFQDLMNSIFKPF
LRKFVLVFF**DDILIY**IKSWKDHVEHVERVLQLLEEKQLYAKIFKCFFGVQEVEY
LGHIVSHEGVKIDPSKIKSIKEWKISTSIKHLRGFLGLTGYYRKFVKNYGRIAAP
LTTLLKKDSFSWTPEATKAFKHLKEAMCQALVVATPDFTKTFIVE**CDAS**GNGI
GVVLMQDERPITFESRLIKGKFLRKDIYEKEMLAILHALKKWRPYLMGRHFKV **RNaseH**
KTDHDSLKYFLEQRLSSEEQQKWVTKMLGYDFEIIYKKGKQNVVADALSKDE
DVEAFLCAISIIQPDWINEAREEWKNDEEVWALIRKLQQYSSTSETFSWKND
SLWYKDHLYLCKNYQLKQKILMEF**H**TSPLGG**H**SRFLKTYHRVKKEFFWDGL
KSDIQKFVAE**C**LV**C**QQNKVATIKTPGLLQPLSIPSQCWEDVSMDFITGLPKSE
GKSVIMVVV**D**RLTKYAHFCTLSHPFKASKVSTAFMETVQKLHGNPKIIVS**D**RD
PIFTGNFWSELFSCLGTQLAHSSSYHPQSDGKT**E**IVNKFLEGYLRCFVSDKQ **INT**
TQWVKLPLAEWWYNTSFHTATKMTPFMALYGYQTPSTTSYLRENSKVQAV
EHHIEHQQQVLQLLKDNLVLAQNRMKQQADQHRSERSFDV**GD**WVFLRLQL
YKKMSLKQAKKDN**KL**SP**KY**Y**GSY**K**V**LQKIGTMAYKLELPAASRL**HPVFHVS**
CLKKVIGDKLPIQTIFQNLTRKEKLYWNLESPKSLVTIFPELDEEGKIIFEPEAV
TETSTRQVRNRSISEYLIKWKNLSTEDSTWEDENFMRKYPELLKREERK…3’ **Chromo**

Additional file 1. Structural features of *Corky* retrotransposon.
Conserved amino acid (single letter code) domains of Reverse Transcriptase (underline), RNaseH, Integrase (underline) and Chromo (underline). In the Reverse Transcriptase two important motives PFGL and DDILIYS are in red. In RNaseH the CDAS motif is pointed in bold. In the integrase the three subdomains are in red: HH-CC; D,DE and G-(D/E)-X_{10-20}-KL-X_2/R/K)-F/Y/W)-X-G-P-(F/Y)-X-(I/V). The HPVFH(V)S motif is showed in bold.
