# Supplementary material for: Corky, a gypsy-like retrotransposon is differentially transcribed in Quercus suber tissues
Source: BMC Res Notes. 2012 Aug 13;5:432. doi: 10.1186/1756-0500-5-432 (PMC3465219; doi:10.1186/1756-0500-5-432)
Supplement: Additional file 2 — Nucleotide sequence of the 5’ LTR from Corky . The repeating sequences motifs are underlined and the two TATA box sequences are boxed. [file 1756-0500-5-432-S2.pdf]

5’

TGTTAGACTCCCTATTATATTAGTGATTACCCCATTACTATTGTAGTTATACCCC
TTATATATATTGTAGTGCCCCTATTACCCCATATTGTTACTATTGTGCCCCATAG
ATGCCCAGTATTGTTATTATTGTGGCCTTATATTATTATTGTTAGGTCGGCTGG
GTTATATTGTGACTCCTAAGACTCCTATATATAGATAGGAGGTCTTGTCATTTG
AATCATCCAAGAACTGTTCATTTATGTAATGTATAGGTGGTTACCTCCTCGAAG
AGGTTGAATTCTAAGCCGTAGAACTATAATATCTATCTATTCTTGAGCTGGTTT
CATAACA

3’

Additional file 2. Nucleotide sequence of the 5’ LTR from *Corky*.
The repeating sequences motifs are underlined and the two TATA box sequences are boxed.
