# Supplementary material for: Corky, a gypsy-like retrotransposon is differentially transcribed in Quercus suber tissues
Source: BMC Res Notes. 2012 Aug 13;5:432. doi: 10.1186/1756-0500-5-432 (PMC3465219; doi:10.1186/1756-0500-5-432)
Supplement: Additional file 3 — Primers used to amplify Corky . [file 1756-0500-5-432-S3.pdf]

Additional file 3. Primers used to amplify *Corky* retrotransposon.

Primer name	Length (bp)	Melting Temperature (ºC)	5'-3' seq
Ap1	22		GTAATACGACTCACTATAGGGC
Ap2	19	65	ACTATAGGGCACGCGTGGT
Ret1 - R	20		TATAACCCAGCCGACCTAAC
Ap1	22	67	GTAATACGACTCACTATAGGGC
Ap2	19		ACTATAGGGCACGCGTGGT
Ret 2- R	22		ATGACCTGTTTGGAGCAGCGAA
Ret 3 - F	19	60	TGCCCCATAGAAGCCCACT
Ret 4 - R	23		AATTGGATTCTCCAAGAAAGAGT
Ret 5 - F	20	60	GTTAGGTCGGCTGGGTTATA
Ret 6 - R	22		TATTGAGATCGAAGAATTTCTC
Ret 7 - F	22	65	CATTGGAAAGAGGACTCAGAAG
Ret 8 - R	20		CCTGAATGCCTTCTCTAATG
Ret 9 - F	22	66	GATAGAAGACCCACCATATTCC
Ret 10 - R	22		TGAGCGAAGATCCAACTTGGTG
Ret 8 - F	22	68	TGAAGAGGAAGAAGAAAGGGAG
Ret 12 – R	21		CAACCCAAGAAATCCTCGCAG
Ret 13 - F	19	57	TTCAACTGAGTCAAATTTC
Ret 12 – R	21		CAACCCAAGAAATCCTCGCAG
Ret 14 -F	23	68	GCAACAAAAGTGGGTCACAAAGA
Ret 10 - R	22		TGAGCGAAGATCCAACTTGGTG
Ret 15 – F	24	65	CTGCGAGGATTTCTTGGGTTGACA
Ret 16 -R	24		GTAACTGCTTCCGGTTCGAATATA
Ret 17 - F	24	65	CACAGGTTTACCCAAATCTGAGGG
Ret 16 - R	24		GTAACTGCTTCCGGTTCGAATATA
Ret 14 -F	23	65	GCAACAAAAGTGGGTCACAAAGA
Ret 18 - R	22		CACAATATAACCCAGCCGACCT
Ret 19 - F	24	68	AAGTTTTGATGTGGGTGATTGGGT
Ret 16 - R	24		TTCGGTGACTACTCCAGGTTCCAA
Ret 19 - F	24	65	AAGTTTTGATGTGGGTGATTGGGT
Ap1	22		GTAATACGACTCACTATAGGGC
Ap2	19		ACTATAGGGCACGCGTGGT
Ret 20 -F	23	66	TCCTCAATCTGATGGGAAAACTG
Ap1	22		GTAATACGACTCACTATAGGGC
Ap2	19		ACTATAGGGCACGCGTGGT

Primers Ret1 and Ret2 as well as Ret19 and Ret20 were used with Ap1 and Ap2 primers for the adaptor supplied with de GenomeWalking Kit, respectively, to amplify 5' and 3' end.
